# *cis*-4-(Tosyl­oxymeth­yl)cyclo­hexa­ne­carboxylic acid

**DOI:** 10.1107/S1600536808003176

**Published:** 2008-02-15

**Authors:** De-Hong Jiang, Zhi-Hua Mao, Hu Zheng

**Affiliations:** a Department of Medicinal Chemistry, West China School of Pharmacy, Sichuan University, Chengdu 610041, People’s Republic of China; b The Center for Testing and Analysis, Sichuan University, Chengdu 610064, People’s Republic of China

## Abstract

The title compound, C_15_H_20_O_5_S, is an inter­mediate in the synthesis of novel amino­carboxylic acid derivatives. The cyclo­hexane ring exhibits a chair conformation. In the crystal structure, adjacent mol­ecules form dimers *via* O—H⋯O hydrogen bonds.

## Related literature

For the use of amino­carboxylic acid derivatives as anti-ulcer agents, see: Hoshina *et al.* (1984 ▶). For related structures, see: Qi *et al.* (2008 ▶); van Koningsveld *et al.* (1972 ▶).
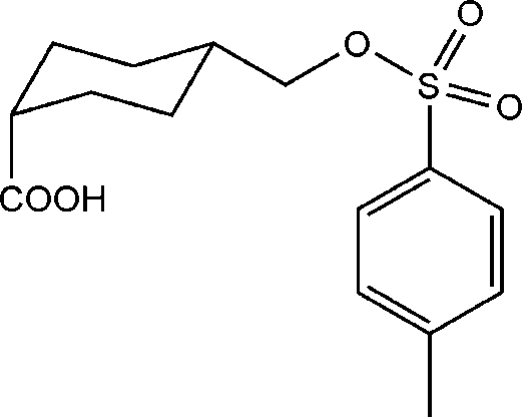


## Experimental

### 

#### Crystal data

C_15_H_20_O_5_S
*M* *_r_* = 312.37
Monoclinic, 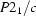

*a* = 12.545 (4) Å
*b* = 10.085 (3) Å
*c* = 12.654 (6) Å
β = 98.05 (3)°
*V* = 1585.1 (10) Å^3^
*Z* = 4
Mo *K*α radiation
μ = 0.22 mm^−1^
*T* = 291 (2) K
0.45 × 0.40 × 0.38 mm

#### Data collection

Enraf–Nonius CAD-4 diffractometer
Absorption correction: none
4142 measured reflections
2931 independent reflections
1794 reflections with *I* > 2σ(*I*)
*R*_int_ = 0.004
3 standard reflections every 250 reflections intensity decay: 0.8%

#### Refinement

*R*[*F* ^2^ > 2σ(*F* ^2^)] = 0.044
*wR*(*F* ^2^) = 0.130
*S* = 1.03
2931 reflections
197 parameters
H-atom parameters constrained
Δρ_max_ = 0.24 e Å^−3^
Δρ_min_ = −0.26 e Å^−3^

### 

Data collection: *DIFRAC* (Gabe *et al.*, 1993 ▶); cell refinement: *DIFRAC*; data reduction: *NRCVAX* (Gabe *et al.*, 1989 ▶); program(s) used to solve structure: *SHELXS97* (Sheldrick, 2008 ▶); program(s) used to refine structure: *SHELXL97* (Sheldrick, 2008 ▶); molecular graphics: *ORTEP-3 for Windows* (Farrugia, 1997 ▶); software used to prepare material for publication: *SHELXL97*.

## Supplementary Material

Crystal structure: contains datablocks global, I. DOI: 10.1107/S1600536808003176/zl2098sup1.cif

Structure factors: contains datablocks I. DOI: 10.1107/S1600536808003176/zl2098Isup2.hkl

Additional supplementary materials:  crystallographic information; 3D view; checkCIF report

## Figures and Tables

**Table 1:** Hydrogen-bond geometry (Å, °)

*D*—H⋯*A*	*D*—H	H⋯*A*	*D*⋯*A*	*D*—H⋯*A*
O5—H5⋯O4^i^	0.82	1.83	2.642 (3)	173

Symmetry code: (i) 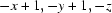.

**Fig. 1.**
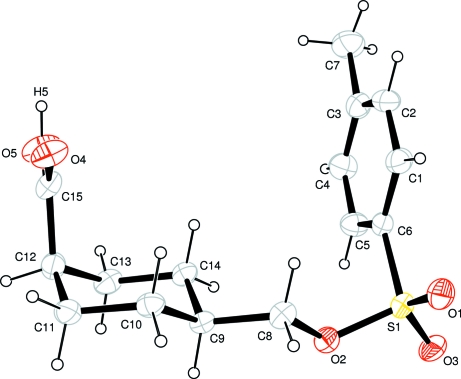
The molecular structure of the title compound with displacement ellipsoids drawn at the 30% probability level.

**Fig. 2.**
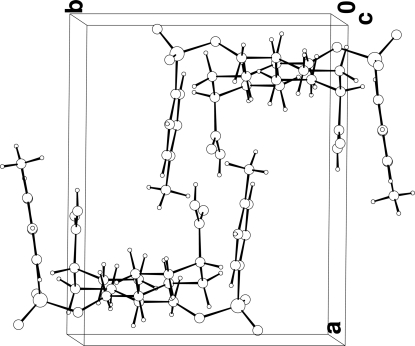
Packing diagram of the title compound.

## supplementary crystallographic information

### Comment

Some aminocarboxylic acid derivatives are used as anti-ulcer agents (Hoshina *et al.*, 1984). To find new anti-ulcer agents, a series of *trans*/*cis*-cyclohexanecarboxylic acid derivatives were designed and synthesized.

In this paper, we want to report the synthesis and structure of the title compound, *cis*-4-(tosyloxymethyl)cyclohexanecarboxylic acid.

The cyclohexane ring exhibits a chair conformation and the cyclohexane C—C bond lengths and C—C—C endocyclic angles are in the range found for similar compounds (van Koningsveld, 1972) (Fig.1). They agree well with those of *trans*-4-(tosyloxymethyl)cyclohexanecarboxylic acid (Qi *et al.*, 2008).

In the crystal structure, two molecules form centrosymmetric dimers *via* O—H···O hydrogen bonds (Fig. 2).

### Experimental

*cis*-4-(Methoxycarboxyl)cyclohexanemethanol (10 mmol), pyridine (11 mmol) and a small amount of 4-dimethylaminopyridine were dissolved in dichloromethane (20 ml), then *p*-toluenesulfonyl chloride (11 mmol) was added dropwise with vigorous stirring at room temperature. After 8 h the reaction was quenched by addition of water and the organic layer separated was evaporated under vacuum, the solid obtained was hydrolyzed in a mixed solution of methanol and aqueous NaOH (11 mmol) for 4 h at 323 K. The title compound was then obtained by acidification with hydrochloric acid followed by recrystallization from ethyl acetate. Colorless crystals suitable for X-ray analysis were obtained by slow evaporation in ethyl acetate at room temperature.

### Refinement

The H atoms were placed in the calculated positions in the riding model approximation with C—H = 0.93 (aromatic-H) and 0.96 (methyl-H), O—H = 0.82 Å (hydroxyl) and with *U*_iso_(H) = 1.2*U*_eq_(aromatic-C) and 1.5*U*_eq_(methyl-C, hydroxyl). Methyl and hydroxyl H atoms were allowed to rotate around the C—C and C—O axis but not to tilt to best fit the experimental electron density.

### Crystal data

**Table:**

C_15_H_20_O_5_S	*F*_000_ = 664
*M**_r_* = 312.37	*D*_x_ = 1.309 Mg m^−^^3^
Monoclinic, *P*2_1_/*c*	Mo *K*α radiation λ = 0.71073 Å
*a* = 12.545 (4) Å	Cell parameters from 43 reflections
*b* = 10.085 (3) Å	θ = 4.4–7.3º
*c* = 12.654 (6) Å	µ = 0.22 mm^−^^1^
β = 98.05 (3)º	*T* = 291 (2) K
*V* = 1585.1 (10) Å^3^	Block, colourless
*Z* = 4	0.45 × 0.40 × 0.38 mm

### Data collection

**Table:**

Enraf–Nonius CAD-4 diffractometer	*R*_int_ = 0.004
Radiation source: fine-focus sealed tube	θ_max_ = 25.5º
Monochromator: graphite	θ_min_ = 1.6º
*T* = 291(2) K	*h* = −15→15
ω/2θ scans	*k* = 0→12
Absorption correction: none	*l* = −6→15
4142 measured reflections	3 standard reflections
2931 independent reflections	every 250 reflections
1794 reflections with *I* > 2σ(*I*)	intensity decay: 0.8%

### Refinement

**Table:**

Refinement on *F*^2^	Hydrogen site location: inferred from neighbouring sites
Least-squares matrix: full	H-atom parameters constrained
*R*[*F*^2^ > 2σ(*F*^2^)] = 0.044	*w* = 1/[σ^2^(*F*_o_^2^) + (0.0689*P*)^2^ + 0.1341*P*] where *P* = (*F*_o_^2^ + 2*F*_c_^2^)/3
*wR*(*F*^2^) = 0.130	(Δ/σ)_max_ < 0.001
*S* = 1.03	Δρ_max_ = 0.24 e Å^−^^3^
2931 reflections	Δρ_min_ = −0.26 e Å^−^^3^
197 parameters	Extinction correction: SHELXL97 (Sheldrick, 2008), Fc^*^=kFc[1+0.001xFc^2^λ^3^/sin(2θ)]^-1/4^
Primary atom site location: structure-invariant direct methods	Extinction coefficient: 0.0109 (15)
Secondary atom site location: difference Fourier map	

### Special details

**Table:**

Geometry. All e.s.d.'s (except the e.s.d. in the dihedral angle between two l.s. planes) are estimated using the full covariance matrix. The cell e.s.d.'s are taken into account individually in the estimation of e.s.d.'s in distances, angles and torsion angles; correlations between e.s.d.'s in cell parameters are only used when they are defined by crystal symmetry. An approximate (isotropic) treatment of cell e.s.d.'s is used for estimating e.s.d.'s involving l.s. planes.
Refinement. Refinement of *F*^2^ against ALL reflections. The weighted *R*-factor *wR* and goodness of fit *S* are based on *F*^2^, conventional *R*-factors *R* are based on *F*, with *F* set to zero for negative *F*^2^. The threshold expression of *F*^2^ > σ(*F*^2^) is used only for calculating *R*-factors(gt) *etc*. and is not relevant to the choice of reflections for refinement. *R*-factors based on *F*^2^ are statistically about twice as large as those based on *F*, and *R*- factors based on ALL data will be even larger.

### Fractional atomic coordinates and isotropic or equivalent isotropic displacement parameters (Å^2^)

**Table:**

	*x*	*y*	*z*	*U*_iso_*/*U*_eq_	
S1	0.88825 (5)	1.11227 (6)	0.13440 (6)	0.0571 (2)	
O1	0.86629 (16)	1.13334 (18)	0.24047 (15)	0.0714 (6)	
O2	0.92693 (13)	0.96607 (16)	0.12094 (14)	0.0587 (5)	
O3	0.96662 (14)	1.19132 (18)	0.09296 (16)	0.0734 (6)	
O4	0.61080 (15)	0.4878 (2)	0.08885 (17)	0.0756 (6)	
O5	0.60020 (17)	0.5097 (3)	−0.08567 (17)	0.0921 (7)	
H5	0.5356	0.5095	−0.0812	0.138*	
C1	0.6708 (2)	1.1419 (3)	0.0859 (2)	0.0655 (7)	
H1	0.6689	1.1457	0.1591	0.079*	
C2	0.5771 (2)	1.1563 (3)	0.0140 (3)	0.0768 (9)	
H2	0.5122	1.1706	0.0399	0.092*	
C3	0.5778 (2)	1.1499 (3)	−0.0942 (3)	0.0718 (8)	
C4	0.6745 (3)	1.1276 (3)	−0.1305 (2)	0.0744 (8)	
H4	0.6763	1.1214	−0.2035	0.089*	
C5	0.7684 (2)	1.1143 (3)	−0.0613 (2)	0.0673 (7)	
H5A	0.8331	1.1002	−0.0876	0.081*	
C6	0.7667 (2)	1.1218 (2)	0.0466 (2)	0.0517 (6)	
C7	0.4746 (3)	1.1693 (4)	−0.1705 (3)	0.1080 (12)	
H7A	0.4173	1.1920	−0.1309	0.162*	
H7B	0.4569	1.0887	−0.2094	0.162*	
H7C	0.4843	1.2394	−0.2196	0.162*	
C8	0.8744 (2)	0.8621 (2)	0.1757 (2)	0.0574 (7)	
H8A	0.8005	0.8870	0.1802	0.069*	
H8B	0.9116	0.8509	0.2477	0.069*	
C9	0.87667 (18)	0.7337 (2)	0.11476 (18)	0.0475 (6)	
H9	0.9516	0.7140	0.1065	0.057*	
C10	0.8351 (2)	0.6228 (2)	0.1803 (2)	0.0537 (6)	
H10A	0.8800	0.6170	0.2490	0.064*	
H10B	0.7623	0.6434	0.1926	0.064*	
C11	0.8356 (2)	0.4903 (2)	0.1233 (2)	0.0628 (7)	
H11A	0.9095	0.4643	0.1201	0.075*	
H11B	0.8036	0.4237	0.1644	0.075*	
C12	0.7748 (2)	0.4936 (3)	0.0110 (2)	0.0618 (7)	
H12	0.7914	0.4111	−0.0242	0.074*	
C13	0.8138 (2)	0.6083 (3)	−0.0531 (2)	0.0620 (7)	
H13A	0.8869	0.5905	−0.0659	0.074*	
H13B	0.7687	0.6138	−0.1218	0.074*	
C14	0.81088 (19)	0.7401 (2)	0.00422 (18)	0.0509 (6)	
H14A	0.7369	0.7627	0.0108	0.061*	
H14B	0.8396	0.8090	−0.0373	0.061*	
C15	0.6550 (2)	0.4981 (2)	0.0095 (2)	0.0608 (7)	

### Atomic displacement parameters (Å^2^)

**Table:**

	*U*^11^	*U*^22^	*U*^33^	*U*^12^	*U*^13^	*U*^23^
S1	0.0529 (4)	0.0520 (4)	0.0648 (5)	−0.0071 (3)	0.0025 (3)	−0.0085 (3)
O1	0.0762 (13)	0.0768 (13)	0.0589 (12)	−0.0026 (10)	0.0017 (10)	−0.0190 (9)
O2	0.0526 (10)	0.0551 (10)	0.0697 (12)	−0.0066 (8)	0.0133 (9)	−0.0022 (8)
O3	0.0561 (12)	0.0641 (11)	0.0990 (15)	−0.0186 (9)	0.0074 (10)	0.0001 (10)
O4	0.0562 (12)	0.1058 (16)	0.0645 (13)	−0.0209 (10)	0.0070 (10)	−0.0001 (11)
O5	0.0640 (13)	0.139 (2)	0.0713 (14)	−0.0219 (14)	0.0036 (11)	0.0193 (13)
C1	0.0573 (17)	0.0739 (18)	0.0659 (18)	−0.0063 (14)	0.0111 (15)	−0.0126 (14)
C2	0.0484 (17)	0.091 (2)	0.091 (2)	−0.0003 (15)	0.0110 (16)	−0.0210 (18)
C3	0.0640 (19)	0.0699 (18)	0.077 (2)	−0.0004 (14)	−0.0058 (17)	−0.0145 (15)
C4	0.076 (2)	0.089 (2)	0.0558 (18)	0.0038 (17)	0.0018 (16)	−0.0006 (15)
C5	0.0593 (17)	0.0799 (19)	0.0640 (19)	0.0029 (14)	0.0128 (15)	−0.0006 (14)
C6	0.0525 (15)	0.0462 (13)	0.0558 (15)	−0.0047 (11)	0.0055 (12)	−0.0056 (11)
C7	0.077 (2)	0.130 (3)	0.106 (3)	0.012 (2)	−0.025 (2)	−0.018 (2)
C8	0.0589 (16)	0.0625 (16)	0.0506 (15)	−0.0092 (12)	0.0071 (13)	0.0009 (12)
C9	0.0410 (13)	0.0525 (13)	0.0482 (14)	−0.0033 (11)	0.0035 (11)	0.0020 (11)
C10	0.0464 (14)	0.0613 (15)	0.0517 (14)	−0.0021 (12)	0.0010 (11)	0.0106 (12)
C11	0.0499 (15)	0.0558 (15)	0.082 (2)	0.0018 (12)	0.0072 (14)	0.0111 (13)
C12	0.0616 (17)	0.0529 (14)	0.0721 (19)	−0.0055 (12)	0.0138 (14)	−0.0088 (12)
C13	0.0559 (15)	0.0810 (18)	0.0511 (15)	−0.0119 (14)	0.0144 (13)	−0.0095 (14)
C14	0.0487 (14)	0.0576 (14)	0.0466 (14)	−0.0070 (11)	0.0070 (11)	0.0062 (11)
C15	0.0596 (17)	0.0564 (15)	0.0647 (19)	−0.0165 (13)	0.0023 (15)	0.0002 (13)

### Geometric parameters (Å, °)

**Table:**

S1—O3	1.4218 (18)	C7—H7C	0.9600
S1—O1	1.423 (2)	C8—C9	1.510 (3)
S1—O2	1.5688 (18)	C8—H8A	0.9700
S1—C6	1.759 (3)	C8—H8B	0.9700
O2—C8	1.464 (3)	C9—C14	1.523 (3)
O4—C15	1.217 (3)	C9—C10	1.526 (3)
O5—C15	1.306 (3)	C9—H9	0.9800
O5—H5	0.8200	C10—C11	1.519 (3)
C1—C6	1.380 (4)	C10—H10A	0.9700
C1—C2	1.390 (4)	C10—H10B	0.9700
C1—H1	0.9300	C11—C12	1.516 (4)
C2—C3	1.372 (4)	C11—H11A	0.9700
C2—H2	0.9300	C11—H11B	0.9700
C3—C4	1.374 (4)	C12—C15	1.501 (4)
C3—C7	1.515 (4)	C12—C13	1.532 (4)
C4—C5	1.372 (4)	C12—H12	0.9800
C4—H4	0.9300	C13—C14	1.517 (3)
C5—C6	1.371 (4)	C13—H13A	0.9700
C5—H5A	0.9300	C13—H13B	0.9700
C7—H7A	0.9600	C14—H14A	0.9700
C7—H7B	0.9600	C14—H14B	0.9700
			
O3—S1—O1	119.95 (12)	C8—C9—C10	108.55 (19)
O3—S1—O2	104.28 (11)	C14—C9—C10	110.34 (18)
O1—S1—O2	110.26 (11)	C8—C9—H9	108.4
O3—S1—C6	108.65 (12)	C14—C9—H9	108.4
O1—S1—C6	108.78 (13)	C10—C9—H9	108.4
O2—S1—C6	103.69 (10)	C11—C10—C9	111.2 (2)
C8—O2—S1	117.09 (15)	C11—C10—H10A	109.4
C15—O5—H5	109.5	C9—C10—H10A	109.4
C6—C1—C2	118.7 (3)	C11—C10—H10B	109.4
C6—C1—H1	120.7	C9—C10—H10B	109.4
C2—C1—H1	120.7	H10A—C10—H10B	108.0
C3—C2—C1	121.7 (3)	C12—C11—C10	113.0 (2)
C3—C2—H2	119.2	C12—C11—H11A	109.0
C1—C2—H2	119.2	C10—C11—H11A	109.0
C2—C3—C4	118.1 (3)	C12—C11—H11B	109.0
C2—C3—C7	120.3 (3)	C10—C11—H11B	109.0
C4—C3—C7	121.6 (3)	H11A—C11—H11B	107.8
C5—C4—C3	121.5 (3)	C15—C12—C11	112.6 (2)
C5—C4—H4	119.3	C15—C12—C13	111.4 (2)
C3—C4—H4	119.3	C11—C12—C13	110.9 (2)
C6—C5—C4	119.9 (3)	C15—C12—H12	107.2
C6—C5—H5A	120.1	C11—C12—H12	107.2
C4—C5—H5A	120.1	C13—C12—H12	107.2
C5—C6—C1	120.2 (3)	C14—C13—C12	112.2 (2)
C5—C6—S1	119.5 (2)	C14—C13—H13A	109.2
C1—C6—S1	120.2 (2)	C12—C13—H13A	109.2
C3—C7—H7A	109.5	C14—C13—H13B	109.2
C3—C7—H7B	109.5	C12—C13—H13B	109.2
H7A—C7—H7B	109.5	H13A—C13—H13B	107.9
C3—C7—H7C	109.5	C13—C14—C9	110.81 (19)
H7A—C7—H7C	109.5	C13—C14—H14A	109.5
H7B—C7—H7C	109.5	C9—C14—H14A	109.5
O2—C8—C9	109.29 (19)	C13—C14—H14B	109.5
O2—C8—H8A	109.8	C9—C14—H14B	109.5
C9—C8—H8A	109.8	H14A—C14—H14B	108.1
O2—C8—H8B	109.8	O4—C15—O5	121.8 (3)
C9—C8—H8B	109.8	O4—C15—C12	123.9 (3)
H8A—C8—H8B	108.3	O5—C15—C12	114.3 (3)
C8—C9—C14	112.73 (19)		

### Hydrogen-bond geometry (Å, °)

**Table:**

*D*—H···*A*	*D*—H	H···*A*	*D*···*A*	*D*—H···*A*
O5—H5···O4^i^	0.82	1.83	2.642 (3)	173

Symmetry codes: (i) −*x*+1, −*y*+1, −*z*.
